# Mary Shortridge Mules

**Published:** 1934

**Authors:** 


### MARY SHORTRIDGE MULES, L.M.S.S.A.

Mary Shortridge Mules was a student at the London School of Medicine for Women and the Royal Free Hospital before she came down here as a student at the Royal Infirmary. She qualified in the summer of 1932. For a year after her qualification she assisted her aunts at their private asylum in Devonshire. She then held the post of Assistant Resident Medical Officer at the Babies' Hospital in Manchester for six months, and in the middle of September this year she went to be House Surgeon at Stroud General Hospital, where she developed a spreading cellulitis of the right arm on Saturday, the 6th of October, 1934, and died, in spite of treatment, the following Tuesday morning.

Although a very retiring individual, she had many good qualities and was very hard-working and painstaking, and her clinical work was excellent. She was getting on very well in her profession when her fatal illness started.

